# Pharmacological Undertreatment of Coronary Risk Factors in Patients with Psoriasis: Observational Study of the Danish Nationwide Registries

**DOI:** 10.1371/journal.pone.0036342

**Published:** 2012-04-30

**Authors:** Ole Ahlehoff, Lone Skov, Gunnar Gislason, Jesper Lindhardsen, Søren Lund Kristensen, Lars Iversen, Stine Lasthein, Robert Gniadecki, Tomas Norman Dam, Christian Torp-Pedersen, Peter Riis Hansen

**Affiliations:** 1 Department of Cardiology, Copenhagen University Hospital Gentofte, Hellerup, Denmark; 2 Department of Internal Medicine, Copenhagen University Hospital Roskilde, Roskilde, Denmark; 3 Department of Dermatology, Copenhagen University Hospital Gentofte, Hellerup, Denmark; 4 Department of Dermatology, Aarhus University Hospital, Aarhus, Denmark; 5 Department of Dermatology, Odense University Hospital, Odense, Denmark; 6 Department of Dermatology, Copenhagen University Hospital Bispebjerg, Copenhagen, Denmark; 7 Department of Dermatology, Copenhagen University Hospital Roskilde, Roskilde, Denmark; Policlinico San Donato Milanese, Italy

## Abstract

**Background:**

Patients with psoriasis have increased prevalence of coronary risk factors and limited recent results have suggested that these risk factors are undertreated in patients with psoriasis. This may contribute to the increased risk of cardiovascular diseases observed in patients with psoriasis.

**Objective:**

To examine the pharmacological treatment of coronary risk factors in patients with severe psoriasis treated with biologic agents in a real-world setting.

**Methods and Findings:**

Medical history of patients with severe psoriasis treated with biologic agents in the time period 2007–09 was retrieved from a Danish nationwide registry (DERMBIO). Individual-level linkage of nationwide administrative registries of hospitalizations, concomitant medications, and socioeconomic status was performed to gain insights into the use of pharmacological treatment. A total of 693 patients (mean age 46.1±12.7 years, 65.7% male) with severe psoriasis treated with biologic agents were identified. Hypertension, hypercholesterolemia, and diabetes mellitus were identified in 16.6%, 9.2%, and 6.7% of cases, respectively. Patients with severe psoriasis were significantly less likely to receive cardiovascular pharmacotherapy compared to age, sex, and coronary risk factor matched controls. In psoriatic patients with hypertension 27.7% received no antihypertensive pharmacotherapy. Patients with dyslipidemia received cholesterol-lowering medications in 55.8% of cases and patients with diabetes mellitus received angiotensin converting enzyme inhibitors/angiotensin II receptor blockers and cholesterol-lowering medications in 42.1% and 23.7% of cases, respectively. Similar results were found for the subset of patients with >1 coronary risk factor and for high risk patients with established atherosclerotic disease.

**Conclusion:**

This nationwide study of patients with severe psoriasis demonstrated substantial undertreatment of coronary risk factors. Increased focus on identifying cardiovascular risk factors and initiation of preventive cardiovascular pharmacotherapy in patients with psoriasis is warranted.

## Introduction

Patients with psoriasis have increased prevalence of classical coronary risk factors, including hypertension, hypercholesterolemia, and diabetes mellitus and increased risk of cardiovascular disease, e.g., myocardial infarction and stroke [1], [2], [3], [4], [5], [6], [7]. Screening practices, treatment, and clinical control aimed at coronary risk factors and disease may be inadequate in patients with other chronic diseases [8], [9] and very recent results from a highly selected population of patients participating in phase III randomized trials with ustekinumab, a therapeutic anti-interleukin (IL)-12/IL-23p40 monoclonal antibody, demonstrated marked undertreatment and underdiagnosis of coronary risk factors in patients with psoriasis [10], [11]. Undertreatment of coronary risk factors may contribute to the increased risk of cardiovascular disease in patients with psoriasis. To examine the current practice regarding the pharmacological treatment of coronary risk factors in patients with psoriasis in a real-world setting we examined the pharmacological management of these risk factors in patients with severe psoriasis treated with biologic agents between 2007 and 2009.

## Methods

### Ethics

The study was approved by The Danish Data Protection Agency and the DERMBIO steering committee. Data at the individual case level was made available by the national registers in anonymized form. Registry studies do not require ethical approval in Denmark. The corresponding author had full access to the data and take responsibility for its integrity.

### Data sources

In Denmark all citizens are provided with a unique social security number at birth enabling linkage across registries. Medical history of patients with severe psoriasis selected for treatment with biologic agents was retrieved from a nationwide Danish registry (DERMBIO), a nationwide Danish database of patients with psoriasis treated with biologic agents. [12] Registration of patients with psoriasis treated with biologic agents is mandatory. From DERMBIO information registered on the date of subject database entry, psoriasis activity and severity index score, baseline history of hypertension, hypercholesterolemia and diabetes mellitus, and previous systemic antipsoriatic treatment and biologic treatment were recorded. Patients with psoriasis identified with hypertension, hypercholesterolemia, and diabetes mellitus were age- and sex matched with 4 individuals with corresponding coronary risk factors (hypertension, hypercholesterolemia, and diabetes mellitus) from the general population. All medications dispensed from pharmacies were obtained from the National Prescription Registry (the Danish Registry of Medicinal Product Statistics), wherein all dispensed prescriptions from Danish pharmacies has been recorded since 1995. Comorbidity, including ischemic heart disease (International classification of diseases 10th revision [ICD-10] codes: I20–I25), cerebrovascular disease (ICD-10: I60–I69), peripheral arterial disease (ICD-10: I70–I74), hypertension (ICD-10: I10), hypercholesterolemia (ICD-10: E78), and type II diabetes mellitus (ICD-10: I11) was obtained from the Danish National Patient Register in which all hospital admissions, diagnoses, and invasive procedures have been recorded since 1978. Individual-level linkage with these nationwide administrative registries of hospitalizations and dispensed prescriptions were used to assess treatment of coronary risk factors. Results were summarized as means with standard deviations for continuous data and counts and percentages for categorical data. Differences in pharmacological treatment were assessed by chi-square test. A two-sided p-value <0.05 was considered statistically significant.

### Outcome measures

Prescriptions claimed up to 6 months prior to study inclusion and 6 months after inclusion for drugs with therapeutic indications aimed at coronary risk factors and cardiovascular diseases including platelet inhibitors (anatomical therapeutic chemical classification [ATC] code: B01AC), beta-blockers (C07), angiotensin-converting enzyme inhibitors (ACE-Is)/angiotensin II receptor blockers (ARBs) (C09), calcium antagonist (C08), loop diuretics (C03C), thiazide diuretics (C03A), cholesterol-lowering drugs (C10A), and glucose-lowering drugs (A10).

## Results

A total of 693 patients (mean age 46.1±12.7 years, 65.7% male) with severe psoriasis treated with biologic agents were identified and baseline characteristics of the study population are presented in Table 1. Coronary risk factors, including hypertension, hypercholesterolemia, and diabetes mellitus were identified in 16.6%, 9.2%, and 6.7%, respectively

**Table 1:** Baseline characteristics of patients with psoriasis.

	Patients with psoriasis n = 693
Age/years (mean, standard deviation)	±12.7
Female (%)	238 (34.3)
Male (%)	455 (65.7)
PASI* score (mean, standard deviation)	13.0±8.1
Psoriatic arthritis	85 (12.3)
Established atherosclerotic disease**	6 (0.9)
**Coronary risk factors**	
Hypertension	94/565 (16.6)
Hypercholesterolemia	52/565 (9.2)
Diabetes mellitus	38/565 (6.7)
**Previous systemic psoriasis treatment**	
Methotrexate	469 (67.7)
Psoralen+UVA***	125 (18.0)
Cyclosporine	161 (23.2)
Retinoids	116 (16.7)
Climate therapy	60 (8.7)
**Biologic agents**	
Adalimumab	279 (40.3)
Alefacept	2 (0.3)
Efalizumab	39 (5.6)
Etanercept	264 (38.1)
Infliximab	64 (9.2)
Tocilizumab	2 (0.3)
Ustekinumab	42 (6.1)
Information not recorded	1 (0.1)

* PASI; psoriasis activity and severity index.,

** Established atherosclerosis; ischemic heart disease, cerebrovascular disease, peripheral vascular disease.

*** UVA; ultraviolet A light.

Patients with psoriasis and coronary risk factors received significantly less evidence based pharmacotherapy compared to age, sex, and coronary risk factor matched individuals from the general population (Tables 2 and 3). In psoriatic patients with hypertension 27.7% received no pharmacotherapy. Along this line, patients with dyslipidemia only received cholesterol-lowering medication in 55.8% of cases. Patients with diabetes mellitus received ACE-Is)/ARBs and cholesterol-lowering medication in 42.1% and 23.7% of cases, respectively. Pharmacologic treatment in patents with >1 cardiovascular risk factor and secondary prophylaxis in the small subset of patents with manifest atherosclerotic disease showed a similar pattern of infrequent use of evidence-based cardiovascular pharmacotherapy (Tables 4 and 5). Assessment of pharmacological treatment 6 months after the index date indicated similar patterns of drug use, however beta blocker use generally decreased, e.g., 12.8% and 2.1% for hypertension at baseline and after 6 months, respectively (Tables 3, 4, 5).

**Table 2:** Medical management of coronary risk factors in patients with psoriasis and age- and sex matched controls.

	Patients with psoriasis	Controls	P value
**Hypertension**	**n = 94**	**n = 376**	
ACE-Is/ARBs*	50 (53.2)	235 (62.5)	0.09
Beta-blockers	12 (12.8)	153 (40.7)	<0.001
Calcium antagonists	25 (26.6)	154 (41.0)	0.01
Thiazide diuretics	16 (17.0)	120 (31.9)	0.004
Any anti-hypertensive drug	68 (72.3)	327 (87.0)	<0.001
**Hypercholesterolemia**	**n = 52**	**n = 208**	
Cholesterol-lowering drugs	29 (55.8)	136 (65.4)	0.19
**Diabetes mellitus**	**n = 38**	**n = 152**	
ACE-Is/ARBs*	16 (42.1)	94 (61.8)	0.03
Platelet inhibitors	9 (23.7)	44 (29.0)	0.52
Cholesterol-lowering drugs	9 (23.7)	87 (57.2)	<0.001

* ACE-1; angiotensin-converting enzyme inhibitor, ARB; Angiotensin II receptor blocker.

**Table 3:** Medical management of coronary risk factors.

	Pharmacotherapy at baseline (%)	Pharmacotherapy at 6 months (%)
**Hypertension n = 94**		
ACE-Is/ARBs*	50 (53.2)	47 (50.0)
Beta-blockers	12 (12.8)	2 (2.1)
Calcium antagonists	25 (26.6)	21 (22.3)
Thiazide diuretics	16 (17.0)	14 (14.9)
Any anti-hypertensive drug	68 (72.3)	58 (61.1)
**Hypercholesterolemia n = 52**		
Cholesterol-lowering drugs	29 (55.8)	29 (55.8)
**Diabetes mellitus n = 38**		
ACE-Is/ARBs*	16 (42.1)	14 (36.8)
Platelet inhibitors	9 (23.7)	9 (23.7)
Cholesterol-lowering drugs	9 (23.7)	12 (31.6)

* ACE-1; angiotensin-converting enzyme inhibitor, ARB; Angiotensin II receptor blocker.

**Table 4:** Cardiovascular prophylaxis in patients with >1 coronary risk factor (n = 40).*

	Pharmacotherapy at baseline (%)	Pharmacotherapy at 6 months (%)
ACE-Is/ARBs**	19 (47.5)	25 (62.5)
Beta-blockers	6 (15.0)	2 (5.0)
Platelet inhibitors	10 (25.0)	11 (25.5)
Cholesterol-lowering drugs	17 (42.5)	21 (52.5)

* Coronary risk factor; hypertension, hypercholesterolemia, diabetes mellitus.

** ACE-I; angiotensin-converting enzyme inhibitor, ARB; Angiotensin II receptor blocker.

**Table 5:** Secondary cardiovascular prophylaxis in patients with established atherosclerotic disease (n = 6).*

	Pharmacotherapy at baseline (%)	Pharmacotherapy at 6 months (%)
ACE-Is/ARBs**	3 (50.0)	2 (50.0)
Beta-blockers	2 (33.3)	0 (0.0)
Cholesterol-lowering drugs	4 (66.7)	4 (66.7)
Platelet inhibitors	5 (83.3)	3 (50.0)

* Previous hospitalization with a diagnosis of ischemic heart disease, cerebrovascular disease, or peripheral vascular disease.

** ACE-I; angiotensin-converting enzyme inhibitor, ARB; Angiotensin II receptor blocker.

## Discussion

In a nationwide study of patients with psoriasis treated with biologic agents the proportion of patients with coronary risk factors treated with cardio-protective drugs was generally low and this finding was consistent for all examined coronary risk factors. Also, in the subset of patients with >1 identified cardiovascular risk factor and in those with established atherosclerosis, including ischemic heart disease, cerebrovascular disease, and peripheral artery disease the proportion of patients receiving cardio-protective treatment was low. The results add to recent evidence indicating that patients with psoriasis are subject to inadequate pharmacological treatment of coronary risk factors and the observed undertreatment may contribute to the increased risk of adverse cardiovascular events seen in these patients.

In general, adequate treatment of coronary risk factors, including hypertension, hypercholesterolemia, and diabetes mellitus remains a challenge in clinical practice [13], [14], [15]. It is well-established that psoriasis is associated with increased risk of cardiovascular disease, and increased prevalence of risk factors and shared autoimmune and inflammatory pathways between psoriasis and cardiovascular disease may contribute to this association [1], [2], [3], [4], [5], [6], [7]. This has sparked an ongoing discussion of the need for increased awareness of this risk by treating physicians (general practitioners, dermatologists, cardiologists etc.) and for dedicated guidelines on coronary risk factor management in patients with psoriasis [16]. Moreover, recent studies have examined the risk of cardiovascular events in patients with psoriasis according to the Framingham cardiovascular risk prediction score and documented that a high proportion of patients with psoriasis were at substantially increased risk and making them potential candidates for pharmacological cardiovascular primary prophylaxis [11], [17]. In agreement with these findings, previous results from our group demonstrated that patients with severe psoriasis and/or psoriatic arthritis carried an absolute and relative risk of cardiovascular disease comparable to that of patients with diabetes mellitus [6]. Indeed, recent Danish guidelines recommended that cardiovascular primary prophylaxis in patients with severe psoriasis and/or psoriatic arthritis should correspond to the treatment offered to patients with diabetes mellitus [18]. These Danish guidelines largely mirror recent guidelines of the European League Against Rheumatism on the management of cardiovascular risk in patients with inflammatory arthritis, including psoriatic arthritis [19].

Regardless of augmented scientific focus on the association between psoriasis and increased cardiovascular risk, however, a very recent study of patients with psoriasis participating in phase III randomized trials with ustekinumab found that patients with coronary risk factors were subject to suboptimal pharmacological treatment [11]. The present results from a real-world clinical setting are in agreement with this observation and suggest that such undertreatment may pose a general problem in patients with psoriasis. While the low proportion of patients treated with beta-blockers was expected since beta-blockers may cause psoriatic exacerbations [20], the explanation for low use of other cardio-protective drugs is less apparent. Indeed, the reasons for lack of initiation and/or adherence to cardiovascular pharmacotherapy may be more dependent on inadequate knowledge and implementation of current guidelines by physicians, physician- or patient-driven worries about poly-pharmacy, reliance on non-pharmacological treatments, and other less-defined physician and patient preferences, than on evidence-based factors, e.g., treatment denied because of adverse drug reactions. Irrespective of the underlying reasons, it is an apparent paradox that psoriasis patients selected for treatment with expensive biologic agents and regularly evaluated by physicians (as is well-established in Denmark) seemingly elude treatment of coronary risk factors. Less than one percent of patients in the present study had established atherosclerotic disease and the results thus mainly reflect inadequate cardiovascular primary prophylaxis. On the other hand, our previous study of the prognosis following first-time myocardial infarction in Danish patients with psoriasis indicated that the post myocardial infarction pharmacological treatment (secondary prophylaxis) was adequate and non-differential between patients with and without psoriasis, respectively [21]. However, that study was not powered to address differences in treatment related to psoriasis disease severity and did not specifically focus on the initiation and adherence to cardiovascular secondary prophylaxis.

In 2008 the US National Psoriasis Foundation recommended that all patients with psoriasis should be referred to a general practitioner for cardiovascular risk factor assessment, including family history, annual blood pressure measurement, biennial screening for obesity, and lipid and glucose screening every 2nd or 5th year for patients with or without classical coronary risk factors, respectively [16], [22]. Of note, it has also recently been suggested that patients with psoriasis are inadequately screened for coronary risk factors [10]. The current report of pharmacological undertreatment of risk factors may therefore in fact underestimate the true magnitude of this problem. In addition, it has been suggested that current risk assessment tools, e.g., the Framingham risk score underestimate the true risk in patients with chronic inflammatory disease, e.g., systemic lupus erythematosus [23]. This is further supported by numerous studies demonstrating increased carotid intima-media thickening, endothelial dysfunction, and coronary calcification in patients with psoriasis in the absence of established atherosclerotic disease [24], [25], [26]. Thus the distinction between cardiovascular primary and secondary prophylaxis in these patients may be argued to be somewhat arbitrary.

The following limitations should be acknowledged. The study included patients with severe psoriasis treated with biologic agents and the results may not apply to other subsets of patients with psoriasis, e.g., patients treated with topical treatment or non-biologic systemic anti-inflammatory treatment. Information on non-pharmacological treatment of cardiovascular risk factors was unavailable. Also, information on blood pressure, cholesterol levels, blood glucose levels were absent and we were therefore unable to calculate formal cardiovascular risk scores, e.g., Framingham risk score. We had no information about the reasons for lack of cardiovascular pharmacotherapy.

Important strengths of the present study included the nationwide coverage, the direct comparison with age, sex, and risk factor matched controls, and the contemporary real-world clinical setting. The use of nationwide registries of hospitalization data and dispensed prescriptions from all pharmacies in Denmark where healthcare is readily accessible and essentially free of charge minimized selection bias related to sex, age, socioeconomic status, healthcare insurance status and job situation.

### Conclusion

This nationwide study of patients with severe psoriasis treated with biologic agents demonstrated that a high proportion of patients with identified coronary risk factors received little or no cardio-protective pharmacotherapy. The results indicate a need for increased awareness of cardiovascular risk and cardiovascular risk factor management in these patients. Randomized clinical trials of intensified cardiovascular risk factor management in these patients are urgently needed.
